# Differentiation of Human Embryonic Stem Cells into Neuron, Cholinergic, and Glial Cells

**DOI:** 10.1155/2020/8827874

**Published:** 2020-11-25

**Authors:** Kimia Hosseini, Emilia Lekholm, Aikeremu Ahemaiti, Robert Fredriksson

**Affiliations:** ^1^Department of Pharmaceutical Bioscience, Uppsala University, Sweden; ^2^Department of Neuroscience, Uppsala University, Sweden

## Abstract

Human embryonic stem cells (hESCs) are pluripotent cells, capable of differentiation into different cellular lineages given the opportunity. Derived from the inner cell mass of blastocysts in early embryonic development, the cell self-renewal ability makes them a great tool for regenerative medicine, and there are different protocols available for maintaining hESCs in their undifferentiated state. In addition, protocols for differentiation into functional human neural stem cells (hNSCs), which have the potential for further differentiation into various neural cell types, are available. However, many protocols are time-consuming and complex and do not always fit for purpose. In this study, we carefully combined, optimized, and developed protocols for differentiation of hESCs into adherent monolayer hNSCs over a short period of time, with the possibility of both expansion and freezing. Moreover, the method details further differentiation into neurons, cholinergic neurons, and glial cells in a simple, single step by step protocol. We performed immunocytochemistry, qPCR, and electrophysiology to examine the expression profile and characteristics of the cells to verify cell lineage. Using presented protocols, the creation of neuronal cultures, cholinergic neurons, and a mixed culture of astrocytes and oligodendrocytes can be completed within a three-week time period.

## 1. Introduction

Human embryonic stem cells (hESCs), which are derived from the inner cell mass (ICM) of blastocysts, are pluripotent cells that can proliferate indefinitely and still maintain their pluripotency and capability to differentiate into cells of all three germ layers: ectoderm, mesoderm, and endoderm [1, 2]. Their limitless capacity for self-renewal along with the potential of differentiation has made hESCs an effective model for regenerative medicines (Klimasnkaya, et al. 2014). Their use is also appreciated in the field of neurodegenerative diseases [3] such as Alzheimer disease [4] and Parkinson disease [5], as well as mental disorders (Brennand, et al. 2011).

Differentiation of hESCs as adherent cultures reveals a striking similarity between *in vitro* differentiation and *in vivo* embryonic formation of the neuroectoderm [6]. Particularly, under correct maintenance conditions, these cells can be programmed to differentiate into neural stem cells (hNSCs), which naturally are capable of self-renewal and can be further generated into lineages such as mixed neuronal cultures, astrocytes, oligodendrocytes, and cholinergic neurons [7]. Each subtype has a potential role in research of neural development and can therefore be used as an *in vitro* model for a variety of biological assays as well as for therapeutic use [8]. The great potential of hESCs for cell therapy has triggered many research groups to optimize different methods for production and maintenance of these cells. However, a precise outcome of cell differentiation is not always easy to control. Variation in density, culture duration, and composition can lead to major differences and hinder the maturation and robust differentiation of stable cell lineages [9]. One reason for variation in cell cultures is the feeder layer used, and hESCs have traditionally been cultured on mouse embryonic feeder (MEF) layers. The MEF cells need to be prevented from multiplying but, at the same time, should remain metabolically active to be able to secrete required signals and cytokines [10] in order to promote target cell proliferation and support undifferentiated growth and pluripotency [11]. Coculturing feeder cells with human hESCs cause other challenges as well [10], such as exposing the cells to potential contaminants. To overcome these difficulties, developments in the production of feeder-independent culture systems have gained traction. These eliminate the inherent biological variability of an undefined component of the culture and could lead to a more reproducible culture system, enabling translational research [12]. Cytokines that maintain pluripotency, self-renewal, and nondifferentiation status of hESCs, usually secreted by the feeding layers, can instead be directly added to the culture. In addition, the use of a feeder-independent system eliminates concerns about cocultures of stem cells and fibroblasts [13], but it does necessitate the knowledge of which cytokines and factors to add and when.

In this paper, we used feeder-independent conditions, with Matrigel as a substrate to maintain hESCs WA09 (H9) provided by WiCell. The protocol which is previously described by [14] allows culturing and maintaining embryonic stem cells using defined parameters with no or minor differentiation until direct induction. A seven-day neural induction resulting in a homogenous population of NSCs from hESCs can be achieved in this manner and can be further differentiated to obtain neurons, astrocytes, and oligodendrocytes with good confluency in no more than three weeks. A protocol for specifically producing adherent cholinergic neurons has also been optimized. Cholinergic cultures derived from hESCs can be used for modeling Alzheimer's disease, but there have been only a few studies detailing successfully differentiation, mainly through the generation of embryoid bodies (EB) [9]. In this study, we have therefore adopted a 14-16-day protocol for cholinergic differentiation with functional cholinergic neurons from adherent NSCs rather than nonadherent cell aggregates, providing cell cultures better suited for *in vitro* studies.

We have compiled a detailed protocol of culturing hESCs H9 cells until terminal differentiation into neurons, cholinergic neurons, astrocytes, and oligodendrocytes. During the optimization process, we have also compared and tested different products made for similar purposes, and the ones yielding better results are discussed. The cell cultures were examined using qRT-PCR, immunocytochemistry, and electrophysiology to provide characterization of valuable genetic markers, morphological evaluation, and functionality.

## 2. Material and Methods

### 2.1. Coating Culture Plates

Based on the WiCell company protocol (http://www.wicell.org), 6-well culture plates were coated using Matrigel™ (Corning life Science, Cat. No. 354230) at 0.5 mg/6-well plate. 11 ml of cold DMED/F-12 medium was placed in a sterile 15 ml conical tube; a Matrigel™ aliquot was retrieved from the freezer, and 1 ml of the cold DMED/F-12 was added to the Matrigel. After the Matrigel had thawed completely, it was transferred to the conical tube and mixed. Immediately, 1 ml/well was dispensed, and the plate was incubated for 1 hour at 37°C, in a CO_2_ incubator.

### 2.2. Thawing and Feeder-Independent Maintenance of H9 Cell

H9 cells, obtained from WiCell, were maintained and expanded in the feeder-independent culture condition using corning hESC-qualified Matrigel as the membrane matrix and mTeSR™1 Medium (Stem Cell Technology, Cambridge Research Park, Waterbeach, UK), as the growth medium. One pluripotent stem cell vial was removed from a -150°C freezer using forceps and placed in a 37°C water bath, without submerging the cap, until there was just a small bit of ice left in the vial. The vial was then removed and sterilized with 70% ethanol. Cells were transferred into a sterile 15 ml conical tube using a 1 ml pipette. MTeSR™1 Medium (11 ml), prewarmed to room temperature, was added drop-wise to cells in the 15 ml conical tube. Cells were centrifuged at 200 × g for five minutes, and the supernatant was aspirated using a sterilized Pasteur pipette. The cell pellet was resuspended in mTeSR™1 Medium resulting in a final volume of 0.5 ml/well (based on recommendations mentioned in the certificate of analysis). Matrigel-coated plates were retrieved from the CO_2_ incubator, and 1.5 ml of complete growth medium was added in each well before 0.5 ml/well of cell suspension was added. In order to disperse the cell equally, plates were moved quickly back and forth and side to side several times before placing them in the incubator. Cells were fed 2 ml/well of complete growth medium daily, based on recommendation from WiCell.

### 2.3. Passaging H9 Cultures

Cells were observed under a microscope on a daily basis and were passaged when the colonies became large or dense, at approximately 70% confluency, with a nonspin method using Versene (Thermo Fisher Scientific, Cat. No. 15040066) and enzyme-free reagent ReleSR™ (Stem Cell Technology, Cat. No. 05872). Although Versene was recommended by the WiCell protocol, healthier aggregates of undifferentiated cells with ReleSR™ were achieved, which minimized the need for manual selection and separation of differentiated colonies. Cells were passaged three times before neural induction.

### 2.4. Induction of Human Neural Stem Cells

High-quality hESCs cultured at 70-80% confluency were passaged in mTeSR™1 Medium as small aggregates and split into Geltrex® LDEV-Free hESC-Qualified (Thermo Fisher Scientific, Cat. No. A1413301) coated 6-well plates at density of 2.5 × 10^5^–3 × 10^5^ cells per well. After 24 hours, when cells reach density of about 20%, neural induction was started by replacing growth medium with complete neural induction medium: Neurobasal medium + neural induction supplement at a 49 : 1 ratio (Thermo Fisher Scientific, Cat. No. A1647801). From days five to seven, the volume of neural induction medium was doubled for efficient nutrition of the cells. Based on Thermo Fisher Scientific, daily change of culture medium is recommended to ensure maximum cell health.

### 2.5. Expansion of hNSCs

On day seven, when hESCs reached 70-80% confluency, they were dissociated using StemPro® Accutase® reagent (Thermo Fisher Scientific, Cat. No. A11105) and split into Geltrex-coated 6-well culture plates at the ratio of 1 : 6. Neural expansion medium was prepared using Neurobasal medium 49 ml+Advanced™ DMEM/F-12 (Thermo Fisher Scientific, Cat. No. 12634) and 49 ml+GIBCO neural induction supplement 1 ml (Thermo Fisher Scientific, Cat. No. A1647801). To prevent cell death, an overnight treatment of 1 *μ*l/ml ROCK inhibitor Y27632 (Sigma-Aldrich, Cat. No. Y0503) was performed.

### 2.6. Cryopreservation of hNSCs

In order to maintain a cell bank, cells were harvested and cryopreserved after two passages, based on Thermo Fisher Scientific recommendations. The medium was aspirated, and 1 ml room temperature StemPro® Accutase® Reagent was added to each well followed by incubation for five minutes at 37°C until detached. Cell suspension was then transferred into a 15-50 ml conical tube. Wells were also washed with 1 ml DPBS to collect any remaining residual cells and were added to the same conical tube. The suspension was mixed using a 1000 *μ*l pipette to break any remaining aggregates. Cells were centrifuged at 300 × g for 4 minutes, and the supernatant was aspirated. The cell pellet was then resuspended in 1 ml neural expansion medium, and cell concentration was determined using countess II FL cell counter (Thermo Fisher Scientific). The cell suspension was diluted with neural expansion medium to 4 × 10^6^ cells/ml, and the same volume of the neural expansion containing 20% DMSO was added into the tube. One ml of the cell suspension was transferred into each cryotube and frozen at -80°C overnight in Nalgene® Mr. Frosty® Freezing Containers (Fisher Scientific, Cat. No. 15-350-50) with isopropanol and transferred the day after to a -150°C freezer.

### 2.7. Differentiation of hNSCs to Neurons, Astrocytes, and Oligodendrocytes

Differentiation started directly after induction of hNSCs. Cells were plated on Geltrex® LDEV-Free hESC-qualified coated 6-well plate in StemPro NSC SFM medium (Thermo Fisher Scientific, Cat. No. A1050901) at a density of 2.5–5 × 10^4^ cells/cm^2^. After two days, the medium was changed to the appropriate differentiation media prepared as recommended by Thermo Fisher Scientific. Neuron differentiation medium: Neurobasal medium 1x, B-27 serum-free supplement 2% (Thermo Fisher Scientific, Cat. No. 17504044) and GlutaMAX-I Supplement 2 mM (Thermo Fisher Scientific, Cat. No. 35050061). Astrocyte differentiation medium: D-MEM 1x (Thermo Fisher Scientific Cat. No. 11995), N2 supplement 1% (Thermo Fisher Scientific, Cat. No. 17502), Glutamax 2 mM, fetal bovine serum 1% (FBS) 1x. Oligodendrocyte differentiation medium: Neurobasal medium 1x, B-27 2%, GlutaMAX 2 mM T3 30 ng/ml (Sigma-Aldrich, Stockholm, Sweden). The medium was completely replaced every second day for a duration of three weeks. Longer culture times resulted in cells detaching from culture vessels.

### 2.8. Differentiation of hNSCs to Cholinergic Neurons

**Figure 1:**
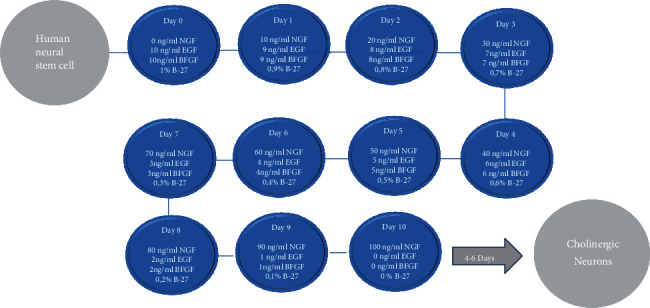
Flow chart of sequential procedure used to produce cholinergic cells derived from human neural stem cells in adherent culture. The protocol involves daily medium change with increasing concentration of NGF and decreasing concentration of EGF, bFGF, and B-27. The medium on day 10 was kept constant for the next four days. The protocol is previously described by [7].

One well of a 6-well plate of hNSCs, at 70% confluency, was split into six wells of a 24-well plate using 2 ml per well of StemPro NSC SFM medium. The cells were allowed to settle for 24 h. A sequential differentiation procedure [7] was then started with a complete replacement of growth medium to differentiation medium containing different concentrations of factors every day (Figure 1). DMEM/F12 (Thermo Fisher Scientific, Cat. No 11320033) medium together with increasing concentration of NGF-beta (Stem Cell Technology, Cat. No. 78092) and reducing concentration of B-27™ Supplement, EGF (Stem Cell Technology, Cat. No. 78006), and BFGF (Stem Cell Technology, Cat. No. 788003.1) was used as complete differentiation medium.

### 2.9. Immunocytochemistry

Cells were seeded on Geltrex-coated coverslip. After completed differentiation, the medium was aspirated, and cells were carefully washed twice with phosphate-buffered saline (PBS) and then fixed in 4% paraformaldehyde (PFA) for 20-30 minutes at room temperature. Wells were then washed three times with PBS to remove PFA completely. Cells were blocked using Supermix (200 ml TBS, 0.5 g gelatin, 1 ml Triton X-100) for 2 h and incubated with primary antibodies at 4°C overnight (Table 1). On day two, cells were washed three times with PBS and incubated with secondary antibody diluted in the Supermix for 2 h. Coverslips were washed twice with PBS and were mounted using ProLong Gold antifade reagent with DAPI (Thermo Fisher Scientific, Cat. No. P36931) and kept at 4°C until visualization. Images were either acquired at the SciLifeLab BioVis Facility (Uppsala University, Sweden) using confocal Zeiss ELYRA S.1 and Zen black software (Zeiss, Oberkochen, Germany) or taken using an Olympus microscope BX53 with an Olympus DP73 camera and the CellSens dimension software. Images were handled using ImageJ (Fiji edition, https://imagej.net/Fiji/).

### 2.10. qRT-PCR

The RNA was extracted using RNAeasy Micro Kit (Qiagen, Cat. No 74004) according to the manufacturer's protocol. The iTag universal SYBR green one-step kit (Bio-Rad Laboratories, Cat. No. 172-5151) was used for qRT-PCR using a thermocycler from Bio-Rad Laboratories. For the total of 10 *μ*l/well of reaction setup, the following components were used: extracted RNA 3-20 ng/*μ*l (depending on the initial RNA concentration), forward and reverse primer 0.5 *μ*M, iScrip reverse transcriptase 0.125 *μ*l, and iTag universal SYBR green reaction mix (2x) 5 *μ*l. The thermal cycling protocol was set up according to the kit recommendation. The experiments were done in three sample replicates of each cell lineage. The master mix without RNA was used as the negative controls. The experiments were repeated 2-3 times. All data was normalized against the housekeeping gene *RPL19*. *β-Tubulin III*, *GALC*, and *GFAP* and *ChAT*, *VGLUT*, and *VIAAT* were used to evaluate characteristics of the cell cultures after three weeks of differentiation (Table 2).

### 2.11. Analysis of qRT-PCR Data

CT-values for each sample were collected, and primer efficiency was calculated for every experiment using LinRegPCR software. *RPLA19* was used as a housekeeping gene, and all the data were normalized against it. Experiments were performed on two biological and three technical replicates of each lineage. The relative mRNA expression was then plotted as mean (SEM) using software GraphPad Prism 5.

### 2.12. Electrophysiology

**Figure 2:**
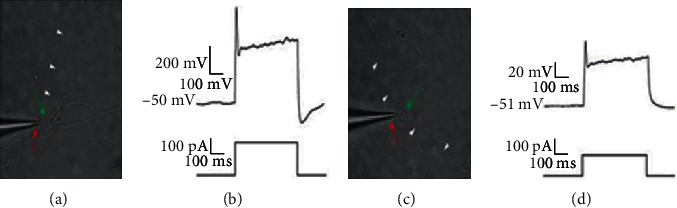
Patch-clamp analysis of electrophysiological properties was performed on neuron. (a) Patched neuron, red arrow indicates the patch pipette while the patched cell is by the green arrow. White triangles indicate the processes of the patched cell. (b) An action potential (upper trace) is elicited by depolarizing current stimulation (pulse duration: 500 ms, range: -10-300 pA, increment: 10 pA). (c) Patched cholinergic neuron with red arrows indicating pipette and green arrow pointing at patched cell. White arrows indicate processes of the patched neuron. (d) Action potential elicited from the patched cholinergic neuron using the same settings as for neurons in (b).

Live cells were identified using a 60x or 20x water immersion objectives (LUMPlan FI, 0.90 numerical aperture (NA), Olympus) for patch clamp visualized on a Zyla sCMOS camera (Andor Technology Ltd). Patch electrodes (6-12 M*Ω*) from borosilicate glass capillaries (GC150F-10 Harvard Apparatus) pulled on a PC-10 gravitational pipette puller (Narishige) contained a K+ based internal solution (in mM): 130 K-gluconate, 40 HEPES, 4 MgCl_2_, 2.16 MgATP, and 3.44 NaGTP, with pH adjusted to 7.2 using 1 M KOH. Liquid junction potential was corrected before each patched neuron. Whole cell patch-clamp recordings were made using a multiclamp 700B amplifier (Axon Instruments) and digitalized with Digidata 1440A (Molecular Devices), low pass filtered at 10 kHz, digitized at 20 kHz, and acquired/analyzed in the WinWCP software (Dr. J. Dempster, University of Strathclyde, Glasgow, UK), Clampfit 10.3 (Molecular Devices, USA), Mini Analysis (Synaptosoft, USA), and Matlab (Mathworks). When the whole cell configuration was achieved, action potentials were induced by current step from -10 pA to 300 pA with an increment of 10 pA (pulse duration 500 ms). Successful firing of an action potential was used (Figure 2).

## 3. Results

In order to characterize and confirm the success of the differentiation protocols, immunocytochemistry, qRT-PCR, and electrophysiology experiments were carried out.

### 3.1. Characterization of Terminally Differentiated Cells

**Figure 3:**
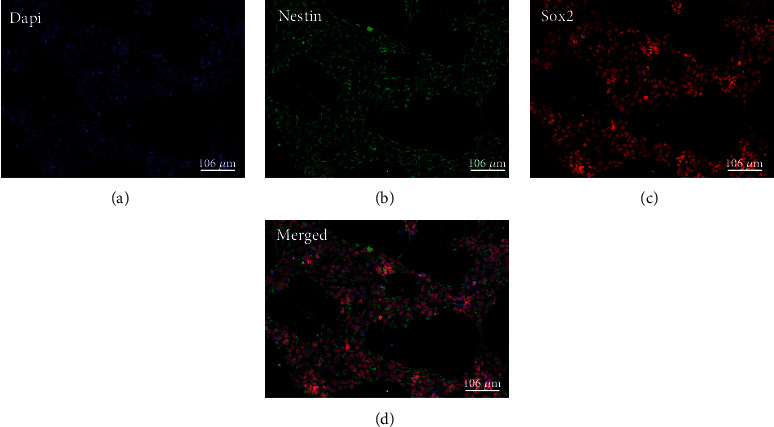
Human neural stem cells differentiated from H9 cells express Sox2 (red) and Nestin (green) proteins. These cells are shown to have progenitor potential. Nuclei stained with DAPI are shown in blue. Scale bar = 106 *μ*m.

**Figure 4:**
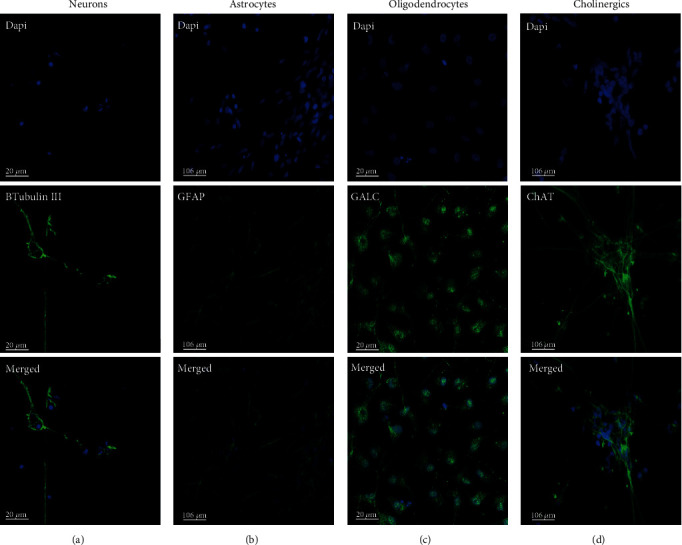
Fluorescence immunocytochemistry on differentiated cell lineages derived from hNSCs: (a) staining of neurons with *β*-tubulin III antibody (scale bar = 20 *μ*m), (b) staining of astrocytes with GFAP antibody (scale bar = 106 *μ*m), (c) staining of oligodendrocytes with GALC antibody (scale bar = 20 *μ*m), and (d) staining of cholinergic neurons with ChAT antibody (scale bar = 106 *μ*m). Nuclei stained with DAPI are shown in blue and targets in green.

hESCs, differentiated into hNSCs in a simple procedure of seven days, were stained using Nestin and Sox2 (Figure 3). These markers were used as a staining control every time new hNSC vial was taken from our cell bank to ensure they were neural progenitors. OCT4 was used as negative control, and no expression was observed (data not shown). Immunocytochemistry was also used to confirm the direction of cell fates into neurons, astrocytes, oligodendrocytes, and cholinergic neurons after three weeks of terminal differentiation (Figure 4). *β*-Tubulin III was used to visualize the presence of neurons, GFAP used for identification of astrocytes, GALC for oligodendrocytes, and ChAT for cholinergic neurons. Each lineage was stained positive with the respective antibody, and significantly reduced expression of Nestin was detected (data not shown) confirming their differentiation.

### 3.2. Gene Expression in Different Lineages

**Figure 5:**
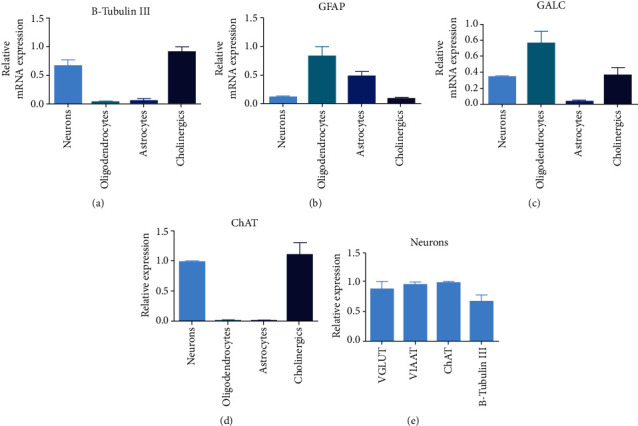
Terminal differentiation cell cultures of neurons, oligodendrocytes, astrocytes, and cholinergic neurons were analyzed using qRT-PCR. In addition, the mixed neuronal cultures were analyzed for neuronal markers. Relative mRNA expressions of different targets were analyzed in the different cultures: (a) mean *β-tubulin III* expression in neurons 0.67 ± 0.11, oligodendrocytes 0.041 ± 0.009, astrocytes 0.04 ± 0.04, and cholinergic 0.9 ± 0.08; (b) mean *GFAP* expression in neurons 0.12 ± 0.01, oligodendrocytes 0.8 ± 0.1, astrocytes 0.5 ± 0.07, and cholinergic 0.01 ± 0.008; (c) mean *GALC* expression in neurons 0.3 ± 0.01, oligodendrocytes 0.7 ± 0.1, astrocytes 0.03 ± 0.01, and cholinergic 0.3 ± 0.08; and (d) mean *ChAT* expression in neurons 0.98 ± 0.01, oligodendrocytes 0.007 ± 0, astrocytes 0.007 ± 0, and cholinergic 1.06 ± 0.1. (e) The neuronal culture was tested for several markers. mRNA expression of *VGULT* mean 0.89 ± 0.16, *VIAAT*0.96 ± 0.05, *ChAT*0.98 ± 0.02, and *β-tubulin III*0.67 ± 0.15 shows mixed culture of glutamatergic, GABA:ergic, and cholinergic neurons. Experiments were performed on two biological and three technical replicates of each lineage. Expression of each gene was normalized against *RPL19*.

To study the gene expression in the different cell lineages, qRT-PCR was used. *β-Tubulin III*, a neuron specific marker, highly expressed in central nervous system [15]. and *ChAT*, a marker for cholinergic neurons in the brain as well as the central nervous system [16], was only expressed in cholinergic neurons and neurons, and almost no expression in oligodendrocytes and astrocytes was found, which shows the effective differentiation of hNSC to neurons and cholinergics (Figures 5(a) and 5(d)). *GALC*, an enzyme that has been used extensively as an oligodendrocytes marker [17], was mostly expressed in oligodendrocytes (Figure 5(c)). Some *GALC* was also found to be expressed in neurons and cholinergic neurons, while very low levels were seen in astrocytes. The highest expression of *GFAP*, an intermediate filament-III protein which is found in astrocytes in the CNS region [18], was observed in oligodendrocytes and astrocytes. Low expression was seen in neurons and cholinergic cultures (Figure 5(b)). The neuronal cultures were a mixture of neuronal types, with expression of *VGLUT* and *VIIAT* in combination with *ChAT* (Figure 5(e)), indicating the precence of glutamatergic, GABA:ergic, and cholinergic neurons in the cultures.

### 3.3. Differentiated Cells Showed Neuron-Like Properties

Protein expression and genetic markers are a valuable tools for lineage verification, but correct physical properties are also needed if the cells are to be used in disease models. Differentiated neurons and cholinergic neurons both had neuron-like morphology and displayed electrophysical properties. Patch-clamp analysis was carried out, and both neuron types were capable of firing action potentials upon stimulation (Figure 2).

## 4. Discussion

Our paper provides a detailed differentiation protocol from hESCs H9 cells to hNSCs and further to neurons, cholinergic neurons, astrocytes, and oligodendrocytes. Matrigel, used as a feeder-independent alternative, provides suitable support to maintain pluripotency and culture of hESCs. hNSCs were induced from hESCs in a simple and reproducible procedure in one week and were verified by staining using neural stem cell markers, Nestin, and Sox2 [19–21]. Compared to other available methods for neural induction in presence of MEF cells, which takes over a month [22, 23] or in absence of MEF cells which usually takes three weeks [8], this protocol manages to produce hNSCs in a short amount of time. hNSCs were then frozen in order to produce a cell bank. Several frozen vials were tested, and all showed to be progenitors and have differentiation potential, making these cells easy to use for further research. We confirmed the success of differentiation protocols using cell staining techniques as well as gene expression analysis by qRT-PCR, and the result indicates a successful differentiation to neurons, astrocytes, oligodendrocytes, and cholinergic neurons. Electrophysiological properties of mixed neuronal cultures and cholinergic neurons were investigated by patch-clamp methods which showed successful action potential generation upon stimulation indicating maturation of the cultures. Presence of *β*-tubulin III staining in neuronal cultures and expression of *β-tubulin III* in neuronal and cholinergic cultures along with its absence in cultures of astrocytes and oligodendrocytes verify these differentiation protocols. Likewise, low expressions of *GFAP* and *GALC* found in both neuronal cultures and cholinergic cultures corroborate the creation of distinctly different cultures. The neuronal cultures were composed of a mixture of neurons. Expression of vesicular inhibitory amino acid transporter (*VIAAT*), vesicular glutamate transporter (*VGLUT*), and *ChAT* quantified by qRT-PCR indicates the presence of GABA:ergic [24], glutamatergic [25], and cholinergic neurons in the culture but with a very low content of glial or astrocytic cells. In this work, we successfully adopted cholinergic differentiation protocols for adherent monolayer culture based on a protocol optimized from nonadherent cultures [7]. Adherent cultures have advantages over nonadherent culture such as easier media changes and easier visualization under a microscope. Adherent cultures can be used for morphological profiling such as cell painting [26] and protein translation assays [27].

We cannot, however, conclude the purity of our astrocytes and oligodendrocytes culture. Based on the gene expression profiles for these cultures, we believe they are a mixed culture of glial cells and oligodendrocytes, perhaps at different stages of maturation. *GFAP* is one of the major components of ependymal cells as well as astrocytes [28]. This intermediate filament protein is expressed in maturing astrocytes but not in mature oligodendrocytes [29], while here, we did see expression of *GFAP* in both the glial and oligodendrocyte cultures (Figure 5(b)). The present protocol does not produce mature oligodendrocytes; however, *GALC* expression nevertheless indicates a high proportion of the culture to be composed of oligodendrocytes. Prolonging the culture time could increase the possibility of more mature oligodendrocytes.

## 5. Conclusion

Protocols presented here can be used to produce cells that can be expanded and frozen as pluripotent cells as well as hNSC. hNSCs derived from H9 cells through a culture set up of seven days show progenitor behavior and are able to differentiate to a mixed neuronal culture of GABA:ergic, glutamatergic, and cholinergic neurons. In addition, cholinergic neurons can be derived from hNESC in adherent culture rather than cell aggregates which makes it easier for downstream applications.

## Figures and Tables

**Table 1:** Details of primary antibody used for fluorescent immunocytochemistry.

Cell lineage	Antibody	Dilution ratio	Source	Cat. No.
NSC	Sox2	1 : 200	Invitrogen	A24354
	Nestin	1 : 200	Abcam	ab6142
	OCT4	1 : 200	Invitrogen	701756
Neurons	*β*-Tubulin III	1 : 200	Abcam	ab18207
Oligodendrocytes	GALC	1 : 200	Abcam	ab232972
Astrocytes	GFAP	1 : 1000	Millipore	MAB360
Cholinergic neurons	ChAT	1 : 100	Millipore	AB144P

**Table 2:** Primers used for qRT-PCR analysis of cultures. *T*: annealing temperature in °C of primers.

Genes	Sequences 5′ to 3′ forward	Sequences 5′ to 3 reverse	*T*
*RPL19*	TGAGGAGAATGAGG	GTACAGGCTGTCAT	55
*β-Tubulin III*	GGCATCTCTTGAGA	GACCTGTACCTGTC	55
*GALC*	GCCAAGCGTTACCATGATTT	TTTCACTCGCTGGAGACCTT	61.4
*GFAP*	CCCTCTGGAGAG	TCCTCCTCGTGGATCTTC	55.1
*ChAT*	AGATGTTCTGCTGC	GAAAAGGATGGTCTCTGG	55.8
*VIAAT*	CTCGGGTCCTTCTGTCCTT	ATCTTGGCCTGGGACTTGTT	61.4
*VGLUT*	GCCAAGCGTTACCATGATTT	TTTCACTCGCTGGAGACCTT	59

## Data Availability

The data used to support the findings of this study are included within the article.
